# Clinical Outcomes after Bilateral Implantation of a Wavefront-Shaping Extended Depth of Focus (EDOF) IOL with Mini-Monovision

**DOI:** 10.3390/jcm13113225

**Published:** 2024-05-30

**Authors:** Yeo Kyoung Won, Sung Ho Choi, Tae-Young Chung, Dong Hui Lim

**Affiliations:** 1 Department of Ophthalmology, Samsung Medical Center, Sungkyunkwan University School of Medicine, 81 Irwon-ro, Gangnam-gu, Seoul 06351, Republic of Korea; 2 First Samsung Eye Clinic, Seoul 06621, Republic of Korea; 3 Renew Seoul Eye Center, Seoul 06181, Republic of Korea; 4 Samsung Advanced Institute for Health Sciences & Technology, Sungkyunkwan University, Seoul 06351, Republic of Korea

**Keywords:** mini-monovision, EDOF IOL, Vivity IOL, visual acuity, optical quality

## Abstract

**Background**: To compare the visual outcomes and optical quality of patients who underwent bilateral implantation of EDOF (AcrySof^®^ IQ Vivity IOL, DFT015) for mini-monovision, trifocal (AcrySof^®^ IQ PanOptix, TNFT00), or monofocal (AcrySof^®^ IQ IOL, SN60WF) IOL. **Methods**: The monocular-corrected and uncorrected distance visual acuities (CDVA and UDVA, respectively) were evaluated postoperatively at 1 and 3 months. The binocular visual acuity by distance, the binocular defocus curve, contrast sensitivity, and patient satisfaction were examined 3 months postoperatively. All patients were asked to complete questionnaires regarding their satisfaction, visual symptoms, and spectacle dependency. **Results**: This study included 178 eyes from 89 patients. The postoperative binocular UDVA did not differ significantly among the three groups. In the defocus curve, the Vivity group showed better visual acuity over a range of far and intermediate (60 cm) than the other two IOLs groups. In near-vision, the PanOptix group showed the best near-vision, and the Vivity group showed significantly better vision than the IQ group. The Vivity group showed contrast sensitivity and optical quality comparable to the IQ group. **Conclusions**: The bilateral implantation of AcrySof^®^ IQ Vivity IOL with the mini-monovision approach provided excellent distance and intermediate visual acuity with good near-vision, resulting in high satisfaction.

## 1. Introduction

Several intraocular lens (IOL) options are currently available for patients who undergo cataract surgery, depending on their particular demands for spectacle independence and tolerance for potential visual disturbances [1,2]. Bifocal and trifocal IOLs have been shown to provide better near-vision than conventional monofocal or extended depth of focus (EDOF) IOLs. However, patients may experience visual disturbances such as blurred vision and photic phenomena, which can be attributed to the distribution of light into multiple foci [1,3]. EDOF IOLs create an elongated focal point to extend the range of vision and decrease photic phenomena by eliminating the overlapping of far and near images, thereby accepting some compromise for near-vision [4,5].

A non-diffractive wavefront-shaping EDOF IOL, the AcrySof^®^ IQ Vivity IOL (Alcon, Fort Worth, TX, USA), was introduced and approved by the US Food and Drug Administration (FDA) in 2020 [6]. This IOL provides an extended focus range with fewer photic phenomena. Although adequate near-vision is a recognized concern, similar to other EDOF IOLs, it helps to improve near-vision with mini-monovision [7,8,9,10,11,12,13,14]. Targeting the non-dominant eye for a slightly myopic refractive error has been successful in improving near-vision with EDOF IOLs while maintaining good binocular distance and intermediate visual acuity [1,8,12,13,14]. As for the Vivity IOL, the effect of mini-monovision has been previously reported [2,12]. However, few data are available regarding the efficacy and stability of AcrySof^®^ IQ Vivity IOL with mini-monovision, compared with other trifocal IOLs.

This study aimed to compare the visual outcomes and optical quality in patients with the bilateral implantation of three IOLs: EDOF IOL for mini-monovision, and trifocal and monofocal IOL.

## 2. Materials and Methods

### 2.1. Study Design

This retrospective, multicenter, non-randomized, comparative study reviewed the electronic medical records of patients who underwent bilateral cataract surgery with implantation of the same type of IOL in each eye between May and September 2021. All patients were asked about their requirements for vision by distance and were provided information about the pros and cons of each IOL. The choice of IOL proposed for implantation was based on their preferences, and patients were divided into three groups according to the implanted IOL type: EDOF, trifocal, or monofocal. The study was approved by the Institutional Review Board (IRB) of the Samsung Medical Center (IRB no. 2021-04-129) and adhered to the tenets of the Declaration of Helsinki. The need for informed consent was waived by the IRB of the Samsung Medical Center (IRB no. 2021-04-129).

### 2.2. Patients

This study included patients who underwent uneventful cataract surgery with the bilateral implantation of one of the three different types of IOLs. The included IOLs were EDOF (Group I; AcrySof^®^ IQ Vivity, DFT015, Alcon, Fort Worth, TX, USA), trifocal (Group 2; AcrySof^®^ IQ PanOptix, TNFT00, Alcon, Fort Worth, TX, USA), and monofocal (Group 3; AcrySof^®^ IQ IOL, SN60WF, Alcon, Fort Worth, TX, USA). Patients were excluded from the study if they had ocular diseases other than cataracts, corneal astigmatism greater than 1.0 D, amblyopia, or previous ocular surgery.

Preoperatively, all patients underwent a complete ophthalmological examination, including corrected and uncorrected visual acuity, manifest refraction, slit-lamp microscopy, and fundus examination. The axial length, refractive power, and anterior chamber depth were measured using the ARGOS^®^ biometer (Alcon, Fort Worth, TX, USA). The IOL power was calculated using the Barrett Universal II formula. The target refraction was emmetropia for PanOptix and IQ monofocal IOL, while mini-monovision was considered for the Vivity IOL group. The definition of mini-monovision has not been firmly established, resulting in varying the range of diopters of mini-monovision across the different studies [7,8,9,10,11,12,13,14,15]. We tried to employ the mini-monovision technique, aiming to achieve the refractive power closest to emmetropia (mostly the first negative values near zero) in the dominant eye, and refractive powers ranging from −0.1 to −0.5 diopters (D) for slight myopia in the non-dominant eye for this study [13,14,15]. The hole-in-the-card test was performed on all patients to detect the dominant eye preoperatively. The test is a sighting dominance technique, in which patients were given a piece of paper with a central circular hole 3 cm in diameter. The patients were asked to hold the paper with both hands and view a target 6 m away through the hole, with both eyes open. Each eye was then occluded in turn. When the dominant eye was covered, the target could not be seen through the hole, while with the nondominant eye being covered, the dominant eye continued to fix the target through the aperture.

### 2.3. Surgical Technique

Surgeries were performed by one of the two experienced surgeons (S.H.C. and T.-Y.C.). All surgical procedures under topical anesthesia were performed using standardized phacoemulsification with a 2.75 mm clear corneal incision. A steep axis corneal incision was created in eyes with corneal astigmatism of more than 0.5D, and a temporal corneal incision was made in eyes with corneal astigmatism of less than 0.5D. Phacoemulsification of the nucleus and bimanual aspiration of the residual cortex were performed using a cataract surgery phacoemulsification device (Centurion Vision System, Alcon, Fort Worth, TX, USA). After the surgery, Lotepro^®^ (0.5% Loteprednol etabonate; Hanlim, Seoul, Republic of Korea) and Gatiflo^®^ (Gatifloxacin; Handok, Seoul, Republic of Korea) were used postoperatively four times a day and tapered over a month. 

### 2.4. Outcome Measures

The patients were evaluated 1 and 3 months postoperatively. All patients underwent corrected and uncorrected distance visual acuity (CDVA and UDVA, respectively) measurements at 5 m using decimal units, and all visual acuities were measured monthly. We converted the reported measurements using decimal units to the logarithm of the Minimum Angle of Resolution (logMAR). 

Three months after surgery, the binocular visual acuity by distance, binocular defocus curve, contrast sensitivity, and patient satisfaction were examined. The defocus curves were plotted by measuring the binocular visual acuity under photopic conditions at 5 m, adding lenses in 0.5D increments from −4.0 to +2.0 D. The contrast sensitivity at 3, 6, 12, and 18 cycles per degree (cpd) was measured using a CSV-1000 chart (Vector Vision, Greenville, OH, USA) under photopic (85 cd/m^2^), mesopic (~3 cd/m^2^), and mesopic with glare conditions. The results were converted into log units for statistical analysis using a specific table for the CSV-1000 [16]. All patients were asked to complete a questionnaire regarding their satisfaction with distance, visual symptoms, and dependency on spectacles for near-, intermediate-, and far-vision. The overall satisfaction was evaluated using 5 levels (very satisfied, satisfied, neither satisfied nor dissatisfied, unsatisfied, and very unsatisfied). To assess the visual symptoms, images and a questionnaire modified from the Quality of Vision questionnaire were used [17]. The patients were shown images and asked to rate the frequency, severity, and bothersome nature of the visual symptoms as none, minimal, moderate, or severe (0, 1, 2, or 3 points, respectively). The mean score was then calculated and spectacle dependence for each distance was investigated using the following scale: 0 = never to 10 = always.

### 2.5. Statistical Analysis

Statistical analyses were performed using the SPSS software (version 18.0; SPSS, Inc., Chicago, IL, USA). The ANOVA was used to perform postoperative comparisons between the three groups. These comparisons were performed using Bonferroni correction.

## 3. Results

The study included 178 eyes of 89 patients who underwent bilateral implantation with one of the three different types of IOL between May and September 2021. A total of 27 patients were included in the Vivity IOL group, 33 in the PanOptix IOL group, and 29 in the IQ monofocal IOL group. Table 1 shows the preoperative patient data. No statistically significant differences among the three IOL groups in terms of age, baseline visual acuity, target, or biometry was found. 

### 3.1. Visual Acuity

Supplementary Tables S1 and S2 show the monocular visual and refractive outcomes 1 and 3 months postoperatively, respectively. Statistically significant differences were observed in the postoperative spherical equivalent (SE) among the groups (*p* = 0.000 and 0.007). Patients in the Vivity group with mini-monovision presented the most myopic refractive outcomes, whereas those in the IQ monofocal IOL group presented hyperopic outcomes at both 1 and 3 months. However, the postoperative monocular UDVA, CDVA, and cylinder diopter at a far distance did not differ significantly among the groups (*p* > 0.05). 

The postoperative monocular visual and refractive outcomes of patients in the Vivity group, subdivided into dominant and non-dominant eyes, are summarized in Supplementary Table S3. The target was closest to emmetropia in the dominant eye and slight myopia (−0.43 D) in the non-dominant eye. The postoperative SE was slightly myopic in both eyes, with higher myopic outcomes in the non-dominant eye compared to the dominant eye at 1 and 3 months postoperatively (*p* = 0.000). Although the postoperative UDVA seemed to be worse in the non-dominant eye regarding the myopic target, no significant difference between the dominant and non-dominant eyes at 1 and 3 months postoperatively was observed (*p* = 0.106 and 0.122, respectively). In addition, no significant difference in the CDVA between the dominant and non-dominant eyes at 1 and 3 months postoperatively was found (*p* = 0.975 and 1.000, respectively). 

Supplementary Table S4 shows the monocular refractive errors in the three groups. No significant differences in the mean absolute error among the groups were found. The binocular uncorrected visual acuity by distance at 3 months postoperatively is shown in Supplementary Table S5. No significant difference in the binocular UDVA among the three IOL groups (*p* = 0.436) was observed. At 80 cm, patients in the Vivity group showed the best binocular vision (*p* = 0.009); at 60 cm, patients in the Vivity group showed significantly better vision than those in the IQ monofocal group (*p* = 0.000). For near distances, patients in the PanOptix group showed better binocular vision than those in the other two IOL groups (*p* = 0.000).

### 3.2. Defocus Curve

**Figure 1:**
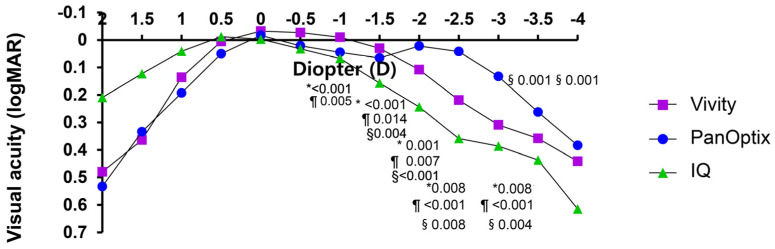
Binocular defocus curves for the three groups. All outcomes were compared among the three groups. Bonferroni correction for multiple comparisons: significant *p*-values (*p* < 0.017) in bold with symbols. *: Vivity versus IQ, §: PanOptix versus IQ, ¶: Vivity versus PanOptix.

The mean defocus curves for the three groups are shown in Figure 1. At a vergence of 0.0 D (distance vision), all eyes of the three groups achieved similar binocular visual acuity results *(p =* 0.813). The patients in the Vivity group (Group 1) showed the best visual acuity over a range of 0.0 D to −1.5 D (corresponding to the visual distances of far to intermediate, 60 cm). The patients in the Vivity group showed significantly better visual acuity than those in the PanOptix group only at −1.0 D and −1.5 D (60 cm to 1 m) for intermediate vision. At vergences of −2.0 D to −4.0 D (25–50 cm), patients in the PanOptix group showed the best vision, followed by those in the Vivity group. For near-vision, the binocular defocus curve of patients in the PanOptix group showed significantly better visual acuity between −2.0 D and −4.0 D (25–50 cm) than of those in the IQ monofocal group. Patients in the PanOptix group also showed significantly better vision at vergences of −2.0 D and −3.0 D (50 cm and 33 cm) than those in the Vivity group. Patients in the Vivity group showed significantly better vision than those in the IQ monofocal group at vergences of −1.5 D and −3.0 D (60 cm and 33 cm).

### 3.3. Contrast Sensitivity

**Figure 2:**
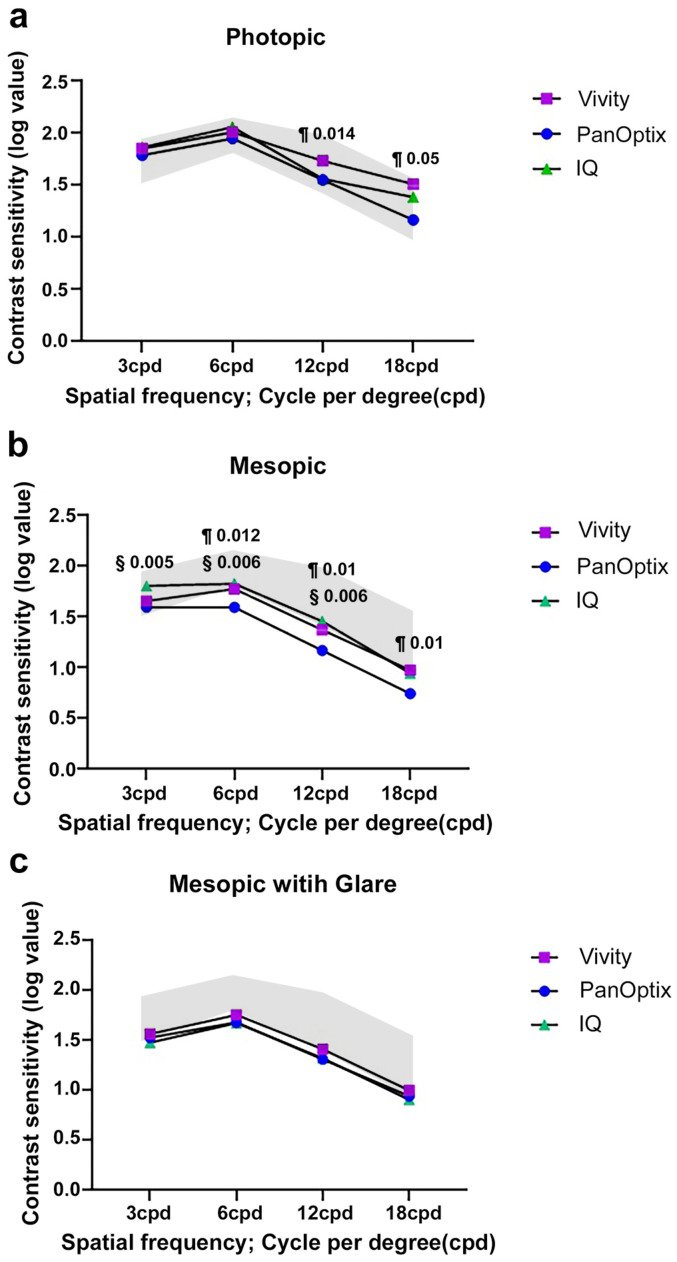
Contrast sensitivity for the three groups. (**a**). Photopic. (**b**). Mesopic with glare off. (**c**). Mesopic with glare on. All outcomes were compared among the three groups. Bonferroni correction for multiple comparisons: significant *p*-values (*p* < 0.017) in bold with symbols. §: PanOptix versus IQ, ¶: Vivity versus PanOptix. Opaque area represents the normal value range.

The binocular contrast sensitivity curves measured at 3 months are shown in Figure 2. In each group, the contrast sensitivity was reduced under mesopic conditions with and without glare compared with photopic conditions. Patients in the Vivity group showed higher contrast sensitivity than those in the PanOptix group at 12 and 18 cpd under photopic conditions. Under mesopic conditions, patients in the Vivity group showed significantly better contrast sensitivity than those in the PanOptix group at 6, 12, and 18 cpd. Patients in the IQ monofocal group showed significantly better outcomes than those in the PanOptix group at 3, 6, and 12 cpd. No significant differences between patients in Vivity IOL and IQ groups were observed. No significant differences were found in any spatial frequency between the groups under mesopic conditions with glare. 

### 3.4. Optical Quality Outcomes

**Figure 3:**
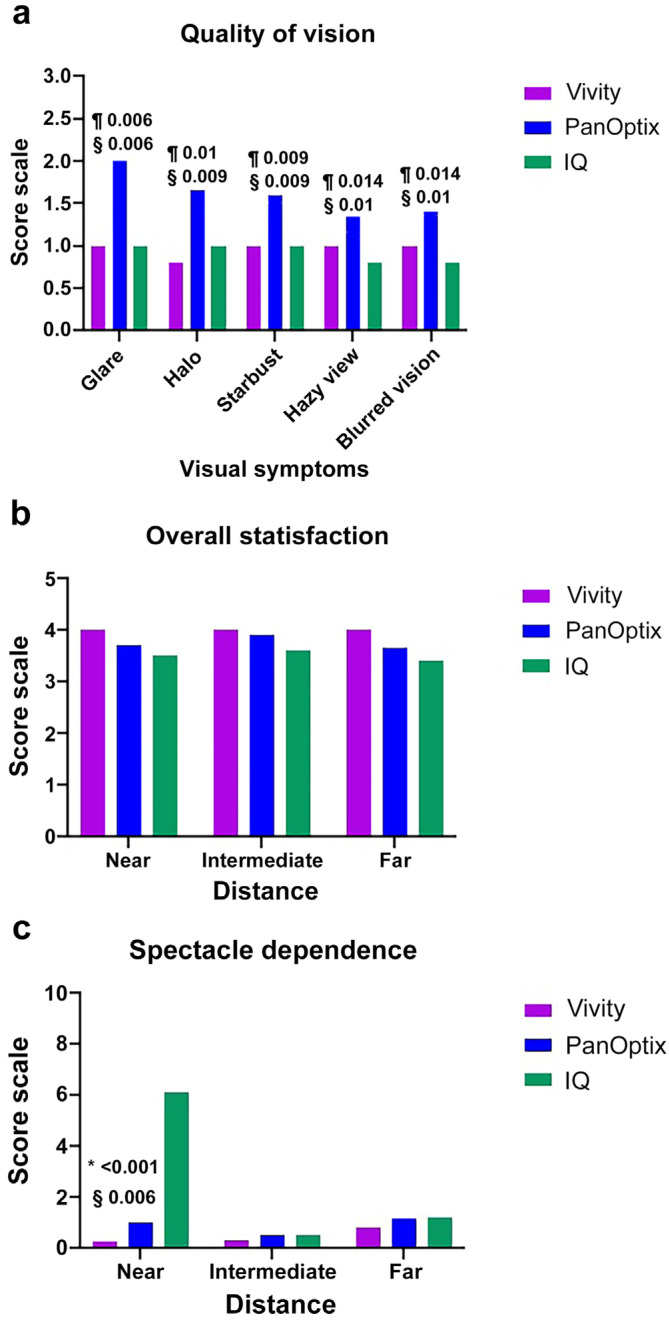
Postoperative questionnaire at 3 months. (**a**) Quality of vision (visual artifacts) questionnaire. (**b**) Overall satisfaction (near, intermediate, and far) questionnaire. (**c**) Spectacle dependence (near, intermediate, and far) in the three groups. All outcomes were compared among the three groups. Bonferroni correction for multiple comparisons: significant *p*-values (*p* < 0.017) in bold with symbols. *: Vivity versus IQ, §: PanOptix versus IQ, ¶: Vivity versus PanOptix. The patients were shown images and asked to rate the frequency, severity, and bothersome visual symptoms as none, minimal, moderate, or severe (0, 1, 2, or 3 points, respectively). The mean score was then calculated. The overall satisfaction was evaluated using 5 levels (very satisfied, satisfied, neither satisfied nor dissatisfied, unsatisfied, and very unsatisfied). Spectacle dependence for each distance was investigated using the following scale: 0 = never to 10 = always.

Figure 3 shows the quality of the visual outcomes for the three groups. First, patients in the PanOptix group reported the highest visual symptom scores (glare, halo, starburst, hazy, and blurred vision) among all three groups (*p* < 0.001). Patients in the Vivity group showed relatively similar scores as those in the IQ monofocal group. No significant difference between the two groups was observed (Figure 3a). Regarding overall satisfaction, patients in the Vivity and PanOptix groups reported relatively similar results for far-, intermediate-, and near-vision, whereas those in the IQ monofocal group tended to have the lowest satisfaction, although no significant difference was observed (Figure 3b). A questionnaire that assessed the postoperative spectacle dependence by distance revealed a significantly higher result at near distance in patients in the IQ monofocal group compared to those of the other groups (*p* < 0.001; Figure 3c).

## 4. Discussion

With the development of various types of multifocal IOLs, patients presenting for presbyopia-correcting cataract surgery are expected to achieve high-quality vision over a wide range of distances without spectacles [18,19,20,21]. However, although trifocal IOLs offer improved near-vision compared to conventional monofocal or EDOF IOLs, they can cause visual disturbances like glare and halo due to light distribution across multiple focal points [1,3]. Therefore, patients who desire better intermediate or near-vision without visual disturbances are opting for EDOF IOL [1,8,22]. Due to EDOF IOL reducing visual disturbances compared to trifocal and providing an extended range of vision compared to monofocal IOL, there have been many studies published on the use of EDOF IOLs. Especially, EDOF IOL with mini-monovision was attempted to overcome the weak near-vision compared to trifocal IOL without compromising the advantages of less dysphotopsia, and good results were reported [23]. A recent study by Ganesh et al. [9] has found that the Tecnis Symfony EDOF IOL (Johnson & Johnson, Santa Ana, CA, USA) targeted for mini-monovision provided excellent outcomes for far- and intermediate-vision with satisfactory near-vision. Cochener et al. [10,11] have shown that the mini-monovision with the Symfony IOL increased the spectacle independence by nearly 9% compared to targeting emmetropia bilaterally. Previous studies [2,22,24,25,26] have also demonstrated the effect of mini-monovision with a non-diffractive EDOF, Vivity IOL. While some recent studies have compared the bilateral implantation of the Vivity IOL with emmetropia and trifocal IOL, other types of EDOF IOL, or Monofocal IOL [12,27,28,29,30,31,32], our study compared the clinical outcomes of the Vivity IOL with mini-monovision trifocal and monofocal IOLs. This study showed that the bilateral implantation of a Vivity IOL with mini-monovision was beneficial for near- and intermediate-vision, with high patient satisfaction.

Although the Vivity IOL showed the most myopic SE postoperatively because of its mini-monovision, the binocular UDVA of 0.0 logMAR or superior in the defocus range of +0.5 D to −0.5 D showed the highest curve among the three groups. This suggested that the Vivity IOL with mini-monovision may be tolerant to low amounts of residual refractive errors. The defocus curve results for bilateral Vivity IOLs have also been reported in several studies [22,24,25,27]. In this study, the binocular visual acuity at far- and intermediate-distances in defocus was comparable with the results of previous studies on Vivity IOL with a target of emmetropia in both eyes [22,25,27,33]. However, the near-vision was more than one line better than that reported in these studies, showing results similar to those previously reported [2,12]. The far and intermediate visual acuity in our defocus curve were also consistent with those of other mini-monovision studies by Amelsfort et al. [22], Coassin et al. [26], and Gundersen et al. [24], and the near-vision slightly differed according to the levels of myopia simulated in the non-dominant eye. In this study, for binocular uncorrected intermediate visual acuity, the Vivity IOL provided the best vision compared to the other two IOLs at 80 cm and better vision than the IQ monofocal IOL at 60 cm. The PanOptix IOL was the best among the three IOLs for near distances (40 and 30 cm). The Vivity IOL with mini-monovision had significantly better near-vision than the IQ monofocal IOL. The IQ monofocal IOL was also targeted for the first negative values closer to emmetropia, but due to the hyperopic postoperative SE, it is challenging to make a direct comparison of near-vision. Thus, the study comparing IQ monofocal IOL with mini-monovision would indeed be necessary in the future.

In the binocular contrast sensitivity test, patients in the Vivity group showed significantly higher values than those in the PanOptix group at high cpd values under both photopic and mesopic conditions. Patients in the PanOptix group generally had a lower contrast sensitivity than those in the other two groups at most spatial frequencies because of its light dissipation [34,35]. In addition, patients in the Vivity group showed comparable results with those in the IQ monofocal IOL group, even in the mesopic condition. No significant differences were observed between the Vivity and IQ monofocal IOLs, similar to the results of Bala et al. [27].

In the questionnaire on visual symptoms, patients in the Vivity group showed similar visual disturbances to those in the IQ monofocal group. The highest scores were noted for patients in the PanOptix group. Regarding overall satisfaction, Panoptix was the highest, followed by Vivity and IQ. However, there were no significant differences between the three groups. This study showed that the Vivity IOL resulted in mild visual symptoms comparable with those of monofocal IOL with high spectacle independence by distance [27,36].

This study had several limitations. Firstly, each IOL group represented a patient population different from a truly randomized comparative sample because of its retrospective design. Specifically, at near distances, the spectacle independence of the Vivity IOLs was higher than that of PanOptix IOL, although the difference was not significant. However, the PanOptix IOL showed better uncorrected binocular near-vision than the Vivity IOL. Therefore, because this study was not a randomized controlled trial, the distribution of the patients per group may not have been the ideal. In this study, the patients who considered near-work less important tended to choose the Vivity IOL rather than the PanOptix IOL, and they were sufficiently satisfied with near-vision with mini-monovision. Secondly, we did not evaluate the reading performance, which reflects the functional near-vision. As Korean letters are much more complex than other letters, and the arms of Asian people are shorter than those of Western individuals, it could be difficult to apply the clinical outcomes in Western people directly. However, no validated Korean version of the reading speed test is available. Lastly, we compared the IQ monofocal IOL without mini-monovision. The IQ monofocal IOL was also targeted for the negative values near zero but less than −0.5, making the IQ monofocal IOL target comparable to the result of Vivity with mini-monovision. However, due to the large mean arithmetic error, the postoperative SE for the IQ monofocal presented hyperopic outcomes due to the retrospective nature of the study. Considering this, for a direct comparison of visual acuity at various distances between Vivity and IQ monofocal, it would be better to correct the hyperopic SE of IQ monofocal IOL or compare both Vivity and IQ monofocal IOLs with mini-monovision. However, our main interest was to determine how much of a difference there is in intermediate- and near-vision between Vivity with mini-monovision and PanOptix, demonstrating that Vivity with monovision does not compromise intermediate-vision compared to PanOptix. We will need to compare it with IQ monofocal with mini-monovision in the future. Further studies with larger sample sizes and longer follow-up periods are required to compare the clinical outcomes of various types of IOLs, including Vivity.

Despite these limitations, this is the first multicenter study to directly evaluate the visual outcomes and optical quality of a non-diffractive EDOF IOL with mini-monovision compared with different types of trifocal and monofocal IOLs. We examined the real-world clinical outcomes in patients who underwent cataract surgery with a bilateral implantation of a Vivity IOL with mini-monovision. We found that the Vivity IOL provided superior intermediate- and near-vision compared to the monofocal IOL, while maintaining contrast sensitivity and visual disturbance similar to that of the IQ monofocal IOL.

In conclusion, the bilateral implantation of the Acrysof IQ Vivity IOL using a mini-monovision approach provided excellent distance and intermediate visual acuity with good near-vision, resulting in high patient satisfaction. As day-to-day activities such as the use of computers, laptops, and tablets increase, EDOF IOLs with mini-monovision would be beneficial for those seeking good intermediate- to near-vision and who do not want visual disturbances.

## Figures and Tables

**Table 1:** Comparison of the preoperative data by IOL group.

	Vivity (Group 1)	PanOptix (Group 2)	IQ Monofocal (Group 3)	*p* Value *
Patients, N	27	33	29	
Age (year)				
Mean ± SD	63.19 ± 3.95	59.55 ± 6.24	72.79 ± 7.45	0.063
Range	[44, 74]	[49, 68]	[50, 86]	
Gender (Female), n (%)	16 (59.3%)	24 (72.7%)	15 (52.7%)	
UDVA (logMAR)				
Mean ± SD	0.43 ± 0.33	0.44 ± 0.45	0.47 ± 0.30	0.950
Range	[0.10, 1.00]	[0.05, 1.40]	[0.09, 1.30]	
Preoperative SE (D)				**0.002**
Mean ± SD	0.42 ± 1.53	−0.56 ± 3.09	0.73 ± 1.83	
Range	[−4.875, 2.75]	[−13.75, 3.5]	[−6.5, 3.5]	
Target (Barrett)	−0.21	−0.06	−0.18	0.054
CDVA (logMAR)				
Mean ± SD	0.13 ± 0.17	0.14 ± 0.14	0.20 ± 0.19	0.195
Range	[0.00, 0.52]	[0.00, 0.82]	[0.00, 0.82]	
AXL (mm)				
Mean ± SD	23.39 ± 0.84	23.82 ± 1.38	23.49 ± 0.87	0.121
Range	[21.94, 28.31]	[20.91, 28.35]	[22.02, 26.71]	
ACD (mm)				
Mean ± SD	3.10 ± 0.30	3.27 ± 0.61	3.04 ± 0.44	0.074
Range	[2.49, 3.95]	[2.38, 4.18]	[2.47, 4.31]	
Flat K (D)				
Mean ± SD	43.79 ± 1.27	43.87 ± 1.62	43.62 ± 1.38	0.634
Range	[40.91, 46.72]	[40.06, 47.12]	[41.82, 46.66]	
Steep K (D)				
Mean ± SD	44.38 ± 1.33	44.62 ± 1.75	44.52 ± 1.39	0.690
Range	[41.70, 47.36]	[40.36, 48.07]	[42.81, 47.15]	

* ANOVA: There were significant differences in preoperative SE between PanOptix and Vivity, and PanOptix and IQ monofocal, respectively. Significant value (*p* Value) is written in bold. SD, Standard deviation; IOL, Intraocular lens; UDVA, Uncorrected distance visual acuity; CDVA, Corrected distance visual acuity; AXL, Axial length; ACD, Anterior chamber depth; K, Keratometry; SE, Spherical equivalent.

## Data Availability

The data presented in the study are available on request from the corresponding author.

## Supplementary Materials

The following supporting information can be downloaded at: https://www.mdpi.com/article/10.3390/jcm13113225/s1, Table S1. Visual and refractive outcomes at 1 month postoperatively. Table S2. Visual and refractive outcomes at 3 months postoperatively. Table S3. Postoperative visual and refractive outcomes in the Vivity group. Table S4. Refractive outcomes at 1, 3 months postoperatively by group. Table S5. Binocular visual acuities by distances at 3 months postoperatively.

## Abbreviations and Acronyms

IOL	Intraocular lens
EDOF	Extended depth of focus
D	Diopter
IRB	Institutional Review Board
CDVA	Corrected distance visual acuity
UDVA	uncorrected distance visual acuity
BCVA	Best corrected visual acuity
Cpd	Cycles per degree
logMAR	Logarithm of the Minimum Angle of Resolution
SE	Spherical equivalent
