# Supplementary material for: Clinical Outcomes after Bilateral Implantation of a Wavefront-Shaping Extended Depth of Focus (EDOF) IOL with Mini-Monovision
Source: J Clin Med. 2024 May 30;13(11):3225. doi: 10.3390/jcm13113225 (PMC11172505; doi:10.3390/jcm13113225)
Supplement: Supplementary file 1 [file jcm-13-03225-s001.zip › jcm-2956219-supplementary.pdf]

Supplementary Table S1. Visual and refractive outcomes at 1 month postoperatively.

	Vivity (Group 1)	PanOptix (Group 2)	IQ monofocal (Group 3)	P value*	Group 1 vs Group 2†	Group 1 vs Group 3†	Group 2 vs Group 3†
**UDVA**	0.07 ± 0.09	0.06 ± 0.08	0.09 ± 0.08	0.107	1.000	0.276	0.138
**CDVA**	-0.00 ± 0.04	0.02 ± 0.05	0.02 ± 0.04	0.055	0.101	0.112	1.000
**Cyl (D)**	-0.44 ± 0.28	-0.51 ± 0.48	-0.64 ± 0.37	0.057	1.000	0.107	0.145
**Postoperative SE(D)**	-0.33 ± 0.42	-0.14 ± 0.32	0.11 ± 0.41	**0.000**	0.123	**0.000**	**0.001**

* ANOVA

†Bonferroni test (p<0.017)

Data are expressed as mean ± standard deviation

UDVA, Uncorrected distance visual acuity; CDVA, Corrected distance visual acuity; Cyl, Cylinder; SE, Spherical equivalent; D, Diopter

Supplementary Table S2. Visual and refractive outcomes at 3 months postoperatively.

	Vivity (Group 1)	PanOptix (Group 2)	IQ monofocal (Group 3)	P value*	Group 1 vs Group 2	Group 1 vs Group 3	Group 2 vs Group 3
**UDVA**	0.07 ± 0.10	0.04 ± 0.06	0.08 ± 0.07	0.064	0.366	1.000	0.086
**CDVA**	-0.01 ± 0.02	0.01 ± 0.03	0.02 ± 0.04	0.054	0.058	1.000	0.501
**Cyl (D)**	-0.46 ± 0.24	-0.45 ± 0.34	-0.66 ± 0.37	0.070	0.169	1.000	0.234
**Postoperative SE(D)**	-0.34 ± 0.45	-0.06 ± 0.33	0.12 ± 0.41	**0.007**	0.107	**0.000**	**0.009**

ANOVA

[†]Bonferroni test (p<0.017)

Data are expressed as mean ± standard deviation

UDVA, Uncorrected distance visual acuity; CDVA, Corrected distance visual acuity; Cyl, Cylinder; SE, Spherical equivalent; D, Diopter

Supplementary Table S3. Postoperative visual and refractive outcomes in the Vivity group.

	Dominant eye	Non-dominant eye	P value*
**Target(Barrett)**	-0.03	-0.43	
**1 Month**			
**Postoperative SE(D)**			
Mean ± SD	-0.09 ± 0.37	-0.56 ± 0.33	**0.000**
Range	[-0.875,0.50]	[-1.50,0.125]	
**UDVA**			
Mean ± SD	0.05 ± 0.09	0.11 ± 0.15	0.106
Range	[-0.18,0.22]	[-0.08,0.40]	
**CDVA**			
Mean ± SD	-0.00 ± 0.05	-0.00 ± 0.43	0.975
Range	[-0.18,0.10]	[-0.08,0.10]	
**3 Month**			
**Postoperative SE(D)**			
Mean ± SD	-0.09 ± 0.41	-0.58 ± 0.36	**0.000**
Range	[-1.125,0.50]	[-1.625,-0.25]	
**UDVA**			
Mean ± SD	0.05 ± 0.09	0.10 ± 0.11	0.122
Range	[0.00,0.30]	[0.00,0.40]	
**CDVA**			
Mean ± SD	-0.00 ± 0.02	-0.00 ± 0.02	1.000
Range	[-0.08,0.00]	[-0.08,0.00]	

* Independence t-test

SD, Standard deviation; UDVA, Uncorrected distance visual acuity; CDVA, Corrected distance visual acuity; SE, Spherical equivalent; D, Diopter

Supplementary Table S4. Refractive outcomes at 1, 3 months postoperatively by group.

	Vivity (Group 1)	PanOptix (Group 2)	IQ monofocal (Group 3)	P value*	Group 1 vs Group 2	Group 1 vs Group 3	Group 2 vs Group 3
**Target(Barrett)**	-0.21	-0.06	-0.18				
**1 Month**							
**Postoperative SE(D)**	-0.33 ± 0.42	-0.14 ± 0.32	0.11 ± 0.41				
**ME(D)**	-0.11 ± 0.35	-0.08 ± 0.34	0.29 ± 0.40				
**MAE(D)**	0.30	0.28	0.36	0.065	1.000	0.198	0.088
**MedAE(D)**	0.225	0.235	0.245				
**3 Month**							
**Postoperative SE(D)**	-0.34 ± 0.45	-0.06 ± 0.33	0.12 ± 0.41				
**ME(D)**	-0.13 ± 0.39	-0.02 ± 0.33	0.30 ± 0.31				
**MAE(D)**	0.30	0.27	0.36	0.375	0.504	1.000	1.000
**MedAE(D)**	0.23	0.23	0.29				

* ANOVA

† Bonferroni test (p<0.017)

MAE, Mean absolute error; ME, Mean arithmetic error; MedAE, Median of absolute error; SE, Spherical equivalent; D, Diopter

Supplementary Table S5. Binocular visual acuities by distances at 3 months postoperatively.

	Vivity (Group 1)	PanOptix (Group 2)	IQ monofocal (Group 3)	P value*	Group 1 vs Group 2	Group 1 vs Group 3	Group 2 vs Group 3
**UDVA**	-0.03±0.07	-0.01±0.09	-0.00±0.09	0.436	1.000	0.779	0.751
**UIVA (80cm)**	-0.01±0.08	0.04±0.09	0.07±0.08	**0.009**	**0.015**	**0.009**	1.000
**UIVA (60cm)**	0.02±0.07	0.03±0.08	0.08±0.11	**0.000**	0.150	**0.000**	0.081
**UNVA (40cm)**	0.22±0.12	0.04±0.09	0.36±0.13	**0.000**	**0.000**	**0.003**	**0.000**
**UNVA (33cm)**	0.31±0.09	0.13±0.11	0.36±0.15	**0.000**	**0.000**	0.023	**0.000**

[*] ANOVA

[†]Bonferroni test (p<0.017)

Data are expressed as mean ± standard deviation

UDVA, Uncorrected distance visual acuity; UIVA, Uncorrected intermediate visual acuity; UNVA, Uncorrected near visual acuity
